# An Explanation

**Published:** 1889-03

**Authors:** Julius G. Schmitt

**Affiliations:** Rochester, N. Y.


### AN EXPLANATION.

Editors Homœopathic Physician:—In your issue of this month, my revered friend, Dr. P. P. Wells, asks for an explanation how I came to quote him as having said: "A high potency will act where a low will." After looking over the proceedings of International Hahnemannian Association for 1886, page 248 and 249, I found that I had misrepresented Dr. P. P. Wells, who had then in substance made the same remarks as in your last edition.

I therefore beg his pardon for my error, made in an off-hand talk in one of our Rochester meetings.

Julius G. Schmitt.

Rochester, N. Y., February 6th, 1889.

